# Growth and Deposition of Au Nanoclusters on Polymer-wrapped Graphene and Their Oxygen Reduction Activity

**DOI:** 10.1038/srep21314

**Published:** 2016-02-22

**Authors:** Tsuyohiko Fujigaya, ChaeRin Kim, Yuki Hamasaki, Naotoshi Nakashima

**Affiliations:** 1 International Institute for Carbon-Neutral Energy Research (WPI-I^2^CNER), Kyushu University, 744 Motooka Nishi-ku, Fukuoka 819-0395, Japan; 2 Department of Applied Chemistry, Graduate School of Engineering, Kyushu University, 744 Motooka Nishi-ku, Fukuoka 819-0395, Japan; 3 JST-CREST, 5 Sanbancho, Chiyoda-ku, Tokyo, 102-0075, Japan

## Abstract

The development of a non-Pt electrocatalyst with a high performance for the oxygen reduction reaction (ORR) is one of the central issues in polymer electrolyte fuel cells science. Au-nanoparticles (Au-NPs) with a diameter of <2 nm are one of the promising substitutes of Pt-NPs; however, it is still a challenge to synthesize such a small-sized Au-NPs with a narrow diameter distribution on a carbon support without using capping agents. We here describe a facile method to deposit uniform Au-NPs (diameter = 1.6 nm and 3.3 nm) on the stacked-graphene (<10 layers) coated with poly[2,2′-(2,6-pyridine)-5,5′-bibenzimidazole] without using any capping agents. The obtained Au-NPs exhibit an excellent ORR activity with the onset potential at −0.11 V and −0.09 V (vs. Ag/AgCl) for 1.6 nm and 3.3 nm, respectively. On the other hand, inhomogeneous Au-NPs with 4.6 nm in average diameter shows the onset potential at −0.15 V (vs. Ag/AgCl).

Despite the inertness of bulk gold, gold nanoparticles (Au-NPs) smaller than 10 nm in diameter were recently found to exhibit a catalytic activity for reactions including the CO oxidation^1^,^2^ and oxygen reduction reactions (ORR)^3^,^4^,^5^,^6^,^7^,^8^,^9^,^10^,^11^,^12^. The unique catalytic activity of the Au-NPs is accounted for by the enhanced adsorption of the reactant molecules on the Au-NP surfaces with a high fraction of surface atoms having low coordination numbers^13^. In particular, the Au-NPs less than 2 nm with a narrow diameter distribution loaded on the conducting supports were reported to have a high catalytic activity for the ORR^14^,^15^. Thus, the Au-NPs with small diameters are considered as one of the alternative catalyst of platinum nanoparticles (Pt-NPs) that have been widely-used as fuel cell electrocatalysts owing to their remarkably high ORR activity^16^,^17^,^18^,^19^,^20^. Notably, different from the Pt-based catalysts, small-sized Au-NPs were revealed to possess remarkable selectivity for the ORR with excellent tolerance to methanol oxidation^9^,^10^,^11^, which means that the methanol poisoning can be ignored, which is ideal for practical applications, especially in the cathode in the direct methanol alkaline fuel cells.

Typically, small-sized Au-NPs with diameters of <2 nm with a narrow diameter distribution are synthesized in bulk solutions using a capping agents^21^. However, the capping agents on the Pt surfaces block the fuel gas access and electron transfer, which leads to serious impair their electroactivity, thereby such Au-NPs immobilized on the conducting supports show sluggish ORR reactions due to their high overpotential^9^,^22^,^23^. In addition, due to their high surface energy, the removal of the capping agents by chemical and/or physical treatments, such as vigorous washing or thermal decomposition, has been known to causes dissolution, aggregation and sintering of the Au-NPs^24^. To overcome these drawbacks, synthetic methods to directly grow the Au-NPs on the conducting supporting materials, such as carbon blacks (CBs), graphene oxide (GO) and carbon nanotubes (CNTs) without using a capping agent have been developed^10^,^14^,^25^, in which the oxidized moieties, such as a carboxyl group, on the carbon materials functions as the binding and nucleation sites of the Au-NPs through the interaction between the negatively charged carboxyl group and positively charged Au ions^26^.

Especially, GO or reduced GO (RGO) have widely been used as the supporting material of metal-NP catalysts and the RGO has been reported to induce a higher catalytic activity due to the synergetic coupling between the RGO and nanoparticles^27^,^28^,^29^,^30^. Indeed, several studies preparing the small Au-NPs without using a capping agent on the RGO have been reported^9^,^10^,^12^,^14^,^25^; however, the composites showing an excellent ORR activity are very limited due to the difficulty in synthesizing uniform small-sized Au-NPs on the supports with a narrow diameter distribution. Such difficulty might be derived from the low melting temperature of the Au-NPs^9^.

Yin *et al.* used hydrazine to reduce GO to RGO to obtain hydrazine-decollated RGO on which Au-NPs (1.8 ± 0.2 nm in diameter) with a high uniform distribution were deposited. The onset potential of the obtained catalyst showed about −0.10 V (vs. Ag/AgCl) and the corresponding current density was ~4.1 mAcm^−2^ at −0.80 V at a 1600 rpm for O_2_-saturated 0.1 M KOH solution. Very recently, Govindhan *et al.* reported a spontaneous growth of the dense dispersion of Au-NPs with an average diameter of 6.8 nm on the RGO based on the electrochemical reduction^10^. The Au-NPs showed onset potential of ~−0.11 V (vs. Ag/AgCl) and the corresponding current density of ~2.3 mAcm^−2^ at −0.4 V at a 1000 rpm under O_2_-saturated 0.1 M KOH solution. However, such oxidized sites are inherently unstable in the high potential region and are readily oxidized causing corrosion, which leads to a low durability of fuel cells^31^. To avoid these crucial drawbacks, it is desirable to design and fabricate functional surfaces that enables the growth of Au-NPs with <2 nm size with high and uniform distribution on the surfaces with highly crystalline graphitic surface.

In this study, we have developed an unique approach to create functional surfaces on the carbon surfaces based on a polymer coating, in which polybenzimidazole (PBI) was used as the polymer since we have already reported that PBI strongly adsorbs to the surfaces of pristine graphitized carbons, such as CNTs and stacked-graphene, and furthermore, they strongly bind to metal ions, such as Pt^32^,^33^,^34^,^35^,^36^,^37^,^38^ and Pd^39^, through the coordination between the metal ions and PBI^34^. The advantage of the method is that the graphitic carbon surfaces are utilized without oxidizing their carbon surfaces. In this study, we describe easy preparation of Au-NPs with ~3.3 nm diameter on the surfaces of pristine graphene and their oxidation reduction reaction (ORR) catalytic activities.

## Results

**Figure 1:**
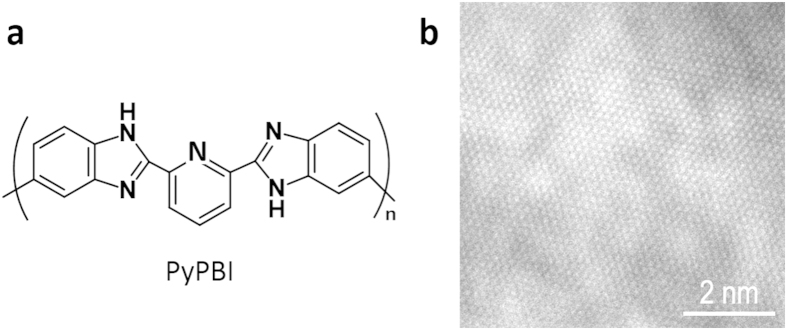
Structural information of PyPBI and s-Graphene/PyPBI. (**a**) Chemical structure of PyPBI, and (**b**) STEM image of the s-Graphene/PyPBI.

**Figure 2:**
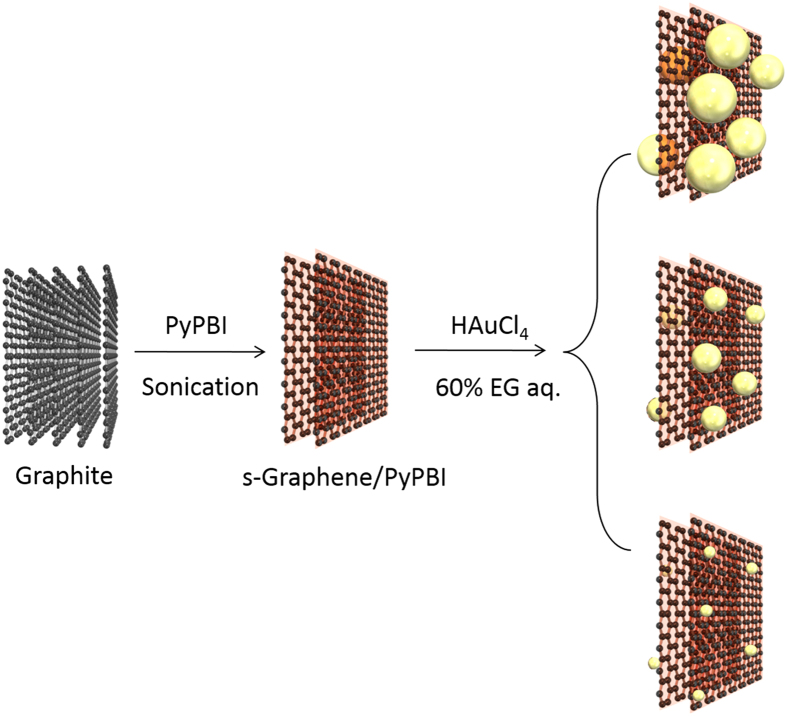
Schematic drawing for the preparation of the s-Graphene/PyPBI/Au_x_. A serious of s-Graphene/PyPBI/Au catalyst were prepared by changing the concentration of the Au salt (1.4 mM; top, 0.7 mM; middle and 0.14 mM; bottom).

We loaded Au-NPs using different feeding amounts of Au salt to i) explore loading efficiency, ii) to tune the size of the Au-NPs, and iii) to study their ORR activities. In this study, pristine graphene having robust two-dimensional sheets of the sp^2^-hybridized carbons was chosen due to its high surface area, enhanced mobility of charge carriers, and high stability^30^,^40^. In our previous study, we reported that non-oxidized stacked-graphene (s-Graphene) was utilized for the loading of the Pt-NPs by the wrapping with PBI^32^. In this study, s-Graphene was exfoliated from graphite and wrapped with poly[2,2′-(pyridine-2,6-diyl)bibenzimidazole-5,5′-diyl] (PyPBI; Fig. 1a) in *N,N*-dimethylacetamide (DMAc) under sonication according to our previous report^32^. As shown in Fig. 1b, evident graphitic structure was observed in the scanning transmission electron microscopy (STEM) images. The advantage of this method is one-step non-destructive exfoliation and wrapping of s-Graphene with PyPBI that served as the binding sites of the metal ions^32^. The Au-NPs were then grew on the surfaces of the PyPBI-wrapped s-Graphene (s-Graphene/PyPBI) using sodium borohydride (NaBH_4_) and three different concentrations (1.4, 0.7 and 0.14 mM) of chloroauric acid (HAuCl_4_) as the reducing agent and Au salt, respectively (Fig. 2).

**Figure 3:**
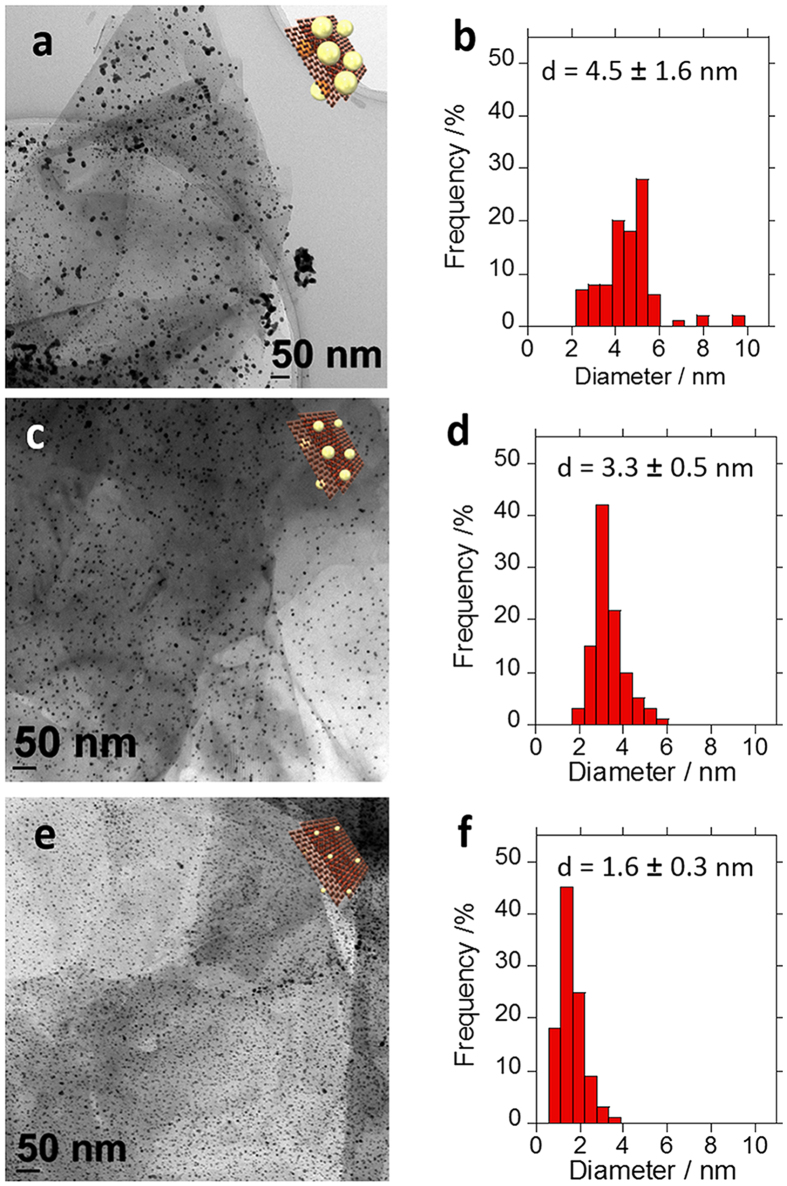
TEM images and the Au diameter histograms of the s-Graphene/PyPBI/Au catalysts. (**a,c,e**) TEM images of (**a**) s-Graphene/PyPBI/Au_4.5_, (**c**) s-Graphene/PyPBI/Au_3.3_ and (**e**) s-Graphene/PyPBI/Au_1.6_. (**b,d,f**) Diameter histograms of (**b**) s-Graphene/PyPBI/Au_4.5_, (**d**) s-Graphene/PyPBI/Au_3.3_ and (**f**) s-Graphene/PyPBI/Au_1.6_ revealed the diameter of Au and density of Au loading were decreased by decreasing the feeding amount of Au salt with maintaining the homogeneous dispersion of Au on s-Graphene/PyPBI. (inset in **a**,**c**,**e**) Schematic drawing of each corresponding catalyst.

**Figure 4:**
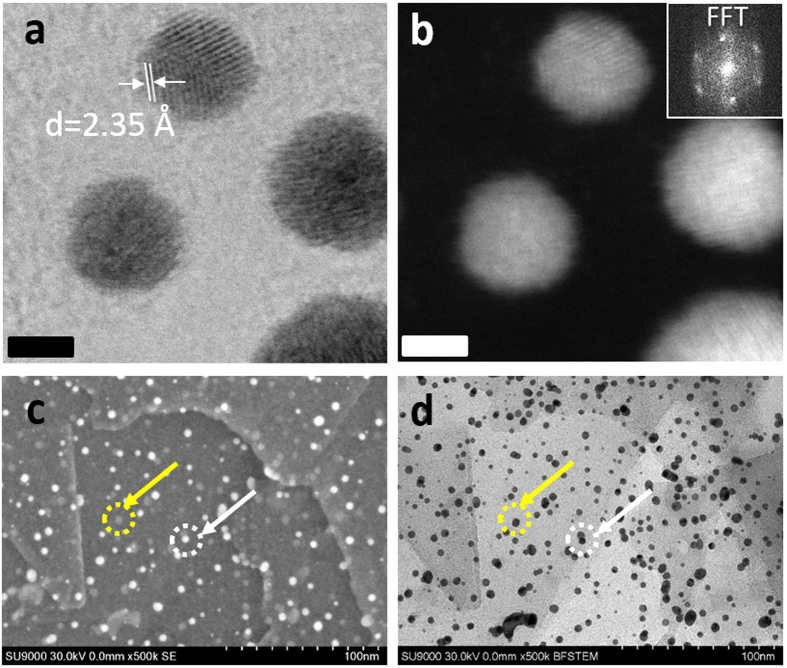
Electron microscope observations of Au-NP on s-Graphene/PyPBI. HR-STEM images in (**a**) bright-field and (**b**) dark-field of the Au_3.3_ show the lattice fringes for the FCC of Au. (inset) FFT pattern for the Au_3.3_. Scale bars in a and b, 2 nm. (**c**) SEM and (**d**) STEM images of Au_3.3_. Yellow and white circles with arrows indicate dark and bright contrast, respectively.

The transmission electron microscope (TEM) images of the prepared three different composites are shown in Fig. 3a,c,e from which the average diameters were determined to be 4.5 ± 1.6, 3.3 ± 0.5 and 1.6 ± 0.3 nm, respectively (denoted as Au_4.5,_ Au_3.3_ and Au_1.6_, respectively). Based on the TEM results, it is evident that we can control the size of the Au-NPs and Au-NCs by simply changing the feeding amounts of the Au salt (Fig. 3b,d,f). In addition, the TEM observation of the Au-NPs and Au-NCs on the s-Graphene/PyPBI at the low-magnification clearly shows homogeneous dispersion at a large area without any agglomeration, especially for Au_3.3_ and Au_1.6_ (see Supplementary Information, Fig. S1). In contrast, large particles were often observed for the Au_4.5_. As shown in Fig. 4a,b, the high-resolution (HR) STEM images revealed that the Au_3.3_ has a highly crystalline structure with the lattice spacing of ~2.35 Å that corresponds to the distance of (111) as the fast Fourier-transform (FFT) analysis (see the inset of Fig. 4d)^41^. Of interest, in the scanning electron microscope (SEM) image, we recognized that ~50% of the Au_3.3_ shows a bright contrast as indicated by the white arrow, and the others showed a dark contrast indicated by the yellow arrow (Fig. 4c). By comparison with the scanning transmission electron microscopy (STEM) image in the same area (Fig. 4d), it was recognized that Au_3.3_ with the bright contrast was loaded on the front side of the s-Graphene and the others were on the back side (for the SEM and STEM images at different magnifications, see Supplementary Information, Fig. S2), which suggests that the Au_3.3_ were loaded on the surfaces of very thin s-Graphene layers.

**Figure 5:**
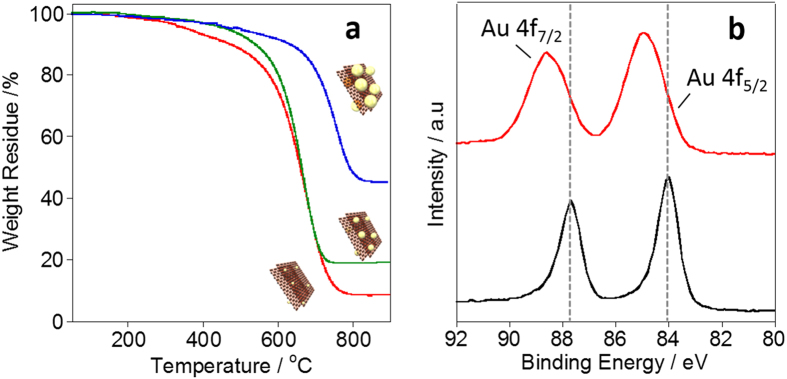
Characterization of the composites. (**a**) TGA curves of s-Graphene/PyPBI/Au_4.5_ (blue line), s-Graphene/PyPBI/Au_3.3_ (green line) and s-Graphene/PyPBI/Au_1.6_ (red line). (**b**) XPS narrow scans of the Au4f region for the s-Graphene/PyPBI + Au (red line) and bulk Au (black line). Indium was used as the substrate.

Loaded weight % of the Au_4.5_, Au_3.3_ and Au_1.6_ on the s-Graphene/PyPBI were determined by thermogravimetric analysis (TGA), which were 45.2, 19.2 and 8.5 wt%, respectively (Fig. 5a). In order to reveal a possible loading mechanism, the s-Graphene/PyPBI was placed in a Au salt solution not containing the reducing agent. The X-ray photoelectron spectroscopy (XPS) of the obtained material (s-Graphene/PyPBI + Au) clearly shows the binding energies at 88.6 (Au 4f 7/2) and 85.0 eV (Au 4f 5/2) that were attributed to the Au(I) (Fig. 5b)^42^. The reduction of Au(III) to Au(I) in the absence of the reducing reagent suggested that the electron transfer from the s-Grapheen/PyPBI to the Au (III) ions occurred. Based on the elemental analysis using XPS, the atomic ratio (Au:N) was estimated to be 1:20. Since the theoretical ratio of Au:N was Au:N = 1:5, it was estimated that the s-Graphene/PyPBI + Au possesses one Au(I) ion per 4 PyPBI units in the absence of the reduction reagent.

We also tested the growth of Au-NPs on non-coated s-Graphene prepared by exfoliation of graphite in the absence of PyPBI at the lowest Au concentration (0.14 mM)^43^, and recognized worm-like Au-NP structures with a broader diameter distribution (11.8 ± 6.0 nm) in the SEM images (see Supporting Information, Fig. S3). The result clearly supports that high and homogeneous dispersion of Au-NPs and Au-NCs with a small size and size distribution deposited on the carbon support is due to the anchoring effect of PyPBI.

**Figure 6:**
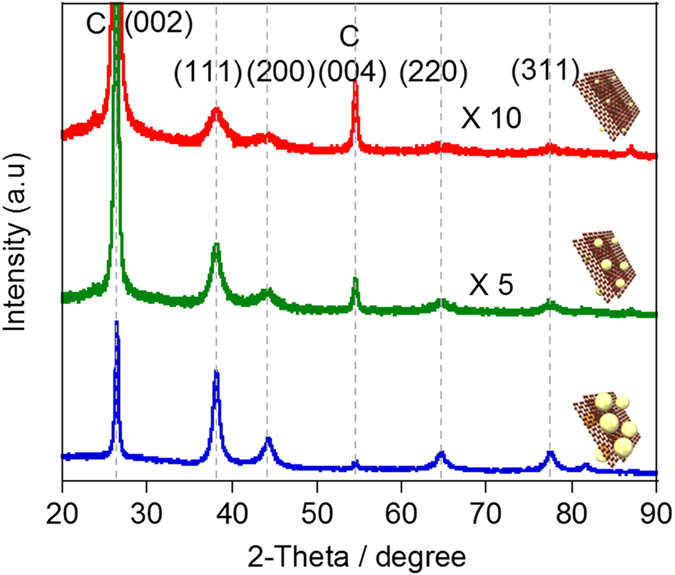
Analysis of the crystalline structure. (**a**) XRD profiles of the s-Graphene/PyPBI/Au_4.5_ (blue line), s-Graphene/PyPBI/Au_3.3_ (green line) and s-Graphene/PyPBI/Au_1.6_ (red line); (inset: schematic drawing of each corresponding catalyst).

**Figure 7:**
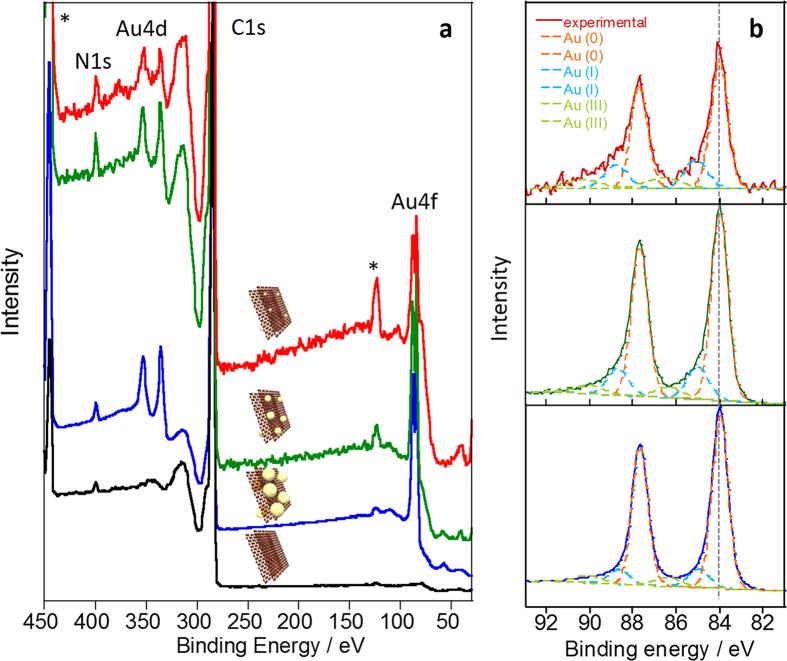
XPS analysis of the composites. (**a**) Wide and (**b**) narrow scans of the XPS of the s-Graphene/PyPBI/Au_4.5_ (blue line), s-Graphene/PyPBI/Au_3.3_ (green line) and s-Graphene/PyPBI/Au_1.6_ (red line). Dotted lines in b are deconvolution curves to estimate the composition ratio of Au (see Supporting Information, Table S2). Peaks with asterisk are from Indium peaks used as the substrate.

Crystalline structure of the Au catalyst was analyzed using X-ray diffraction (XRD) measurements, and the results are shown in Fig. 6, in which we observed four diffraction peaks corresponding to the (111), (200), (220) and (311) planes in the range of 20 to 90° 44. The domain sizes (*τ*) of each phase were calculated using the Scherrer equation (1):


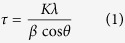


where *K* is the shape factor, *λ* is the X-ray wavelength, *β* is the full width at half maximum (FWHM) of the peaks, and *θ* is the Bragg angle. As a result, decreasing the size as decreasing of the Au concentrations were observed (see Supporting Information, Table S1). It is noted that the *τ* tends to become larger than the average particle size determined by the TEM images since, in the XRD, the signal of a large domain is accentuated and the *τ* is often overestimated^45^. In the XPS of the Au-NPs (Fig. 7a), the Au 4f, Au 4d and N 1s are clearly observed, but the Cl 2p and 2s peaks at ~200 and 271 eV, respectively, were not observed, which supports the reduction of the Au. The composition ratio of the Au(0):Au(II):Au(III) were estimated by the deconvolution of the narrow scans of the Au 4f doublet (4f 7/2 and 4f 5/2) signals in the range of 83~90 eV (Fig. 7b). The composition ratio of Au(0) was found to be decrease as decreasing the size of the particles (see Supporting Information, Table S2). This fact is explained by the increase in the fraction of the surface atoms having low coordination numbers on the support^13^.

**Figure 8:**
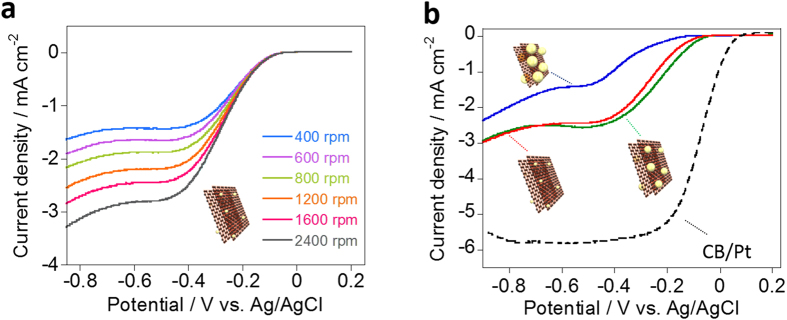
Electrochemical characterizations. (**a**) LSV curves of the s-Graphene/PyPBI/Au_1.6_ for the ORR at the given rotation rates. (**d**) LSV curves of the s-Graphene/PyPBI/Au_4.5_ (blue line), s-Graphene/PyPBI/Au_3.3_ (green line), s-Graphene/PyPBI/Au_1.6_ (red line) and CB/Pt (black line) measured at 1600 rpm in O_2_-saturated 0.1 M KOH solutions at room temperature. Graphics of each corresponding catalyst are presented.

In order to evaluate the catalytic activity of the Au-NCs and Au-NPs, liner sweep voltammetry (LSV) measurements at the rotating speeds of 400, 600, 800, 1600 and 2400 rpm using a rotating disk electrode (RDE) were carried out in N_2_- and O_2_-saturated 0.1 M KOH solutions in the potential range of −0.9–0.2 V (vs. Ag/AgCl). Increasing the limiting current densities as increasing the rotating rates were observed for the Au_1.6_ as shown in Fig. 8a. Similar tendency was observed for both Au_4.5_ and Au_3.3_ (see the Supplementary Information, Fig. S4). As shown in Fig. 8b, the onset potentials of the ORR at 1600 rpm are –0.15, –0.09 and –0.10 V (vs Ag/AgCl) for Au_4.5_, Au_3.3_ and Au_1.6_, respectively. A similar trend was reported for Au-NPs prepared by a different manner^46^,^47^,^48^. Notably, the observed onset potential of −0.09 V for the Au_3.3_ is classified into the lowest overpotential ever reported (for Au-NPs on RGO, onset potential = −0.11 V vs. Ag/AgCl)^10^.

## Discussion

As can be seen in the TEM images in Fig. 3, homogeneous Au-NCs and Au-NPs with a narrow diameter distribution were successfully deposited on the s-Graphene/PyPBI, especially for Au_1.6_ and Au_3.3_. Such result indicated that, at a low concentration of the Au ion, Au nucleation step is significantly faster than the growth step, which leads to uniform small-sized Au nanometals, while the growth step becomes faster at a high concentration and the larger Au-NPs were formed. However, the loading efficiency of the Au nanometal were are almost quantitative for all the composites (Fig. 5a). As proved in XPS (Fig. 5b), such an effective loading was realized due to the effective coordination between the benzimidazole moiety and Au ions similar to the growth mechanism of the Pt-NPs and Pd-NPs onto the PyPBI-wrapped carbon materials^32^,^37^,^38^. Therefore, the fast nucleation step is explained by a coordination-driven mechanism. Indeed, the formation of large particles in the absence of the PyPBI-wrapping (see Fig. S3) even at the lowest concentration (0.14 mM) supported that the growth step became faster in the absence of PyPBI.

In order to consider the mechanism of the uniform and homogeneous loading of the Au-NCs and Au-NPs on the s-Graphene/PyPBI, the density of the clusters (particles) were evaluated. It was calculated that, for Au_1.6_, Au_3.3_ and Au_4.5_, their densities were 0.65, 0.20 and 0.06 particles/100 nm^2^, respectively, which are much smaller than the previous report by Yin *et al.* (ca. 2.8 particles/100 nm^2^)^9^. Decreasing the particle density as increasing of the concentration of Au salt indicated that the growth step of the Au-NCs and Au-NPs at the higher concentrations was faster than the nucleation step. Thus, the regulation of the concentration of the Au salt is crucial to fabricate Au-NCs and Au-NPs with a small diameter with narrow diameter distribution.

In this study, both Au_1.6_ and Au_3.3_ showed a similar leveled high ORR activity (Fig. 8b), which suggested that that smaller Au-NPs is not always superior for the ORR activity. In the Koutecky-Levich (K-L) plots at various electrode potentials based on the LSV curves^46^, similar trend was observed and the number of electrons for the ORR for Au_4.5_, Au_3.3_, and Au_1.6_ were 1.7, 2.5 and 2.4, respectively (see Supporting Information, Table S3). Such a non-linear relationship between the size of Au-NCs and Au-NP and the ORR activity was also observed in a previous literature^9^.

In conclusion, highly dispersed small and uniform Au-NCs and Au-NPs were successfully grown on the s-Graphene/PyPBI surfaces with the aid of the strong anchoring effect of the PyPBI without using any capping regents. The coating with the PyPBI enabled non-distractive modification of the surfaces of the s-Graphene as a growth sites for the Au-NCs and Au-NPs. The small Au-NCs and Au-NPs having 1.6 nm and 3.3 nm in diameter, respectively, were found to show a higher ORR activity than that of the larger Au-NPs (Au_4.5_) probably due to a high fraction of the surface atoms having low coordination numbers.

## Methods

### Materials

Isopropanol, ethylene glycol (EG), DMAc, potassium hydroxide, HAuCl_4_ and NaBH_4_ were purchased from Wako Pure Chemical Industries, Ltd., and used as received. PyPBI was prepared according to a previous paper^38^. Graphite (average diameter; 50 μm) was kindly provided by the Ito Graphite Co., Ltd.

### Measurements

The size and distribution of the Au-NPs were measured using a TEM (JEM-2010, JEOL) at a 120-kV acceleration voltage. A copper grid with a carbon support (Okenshoji Co., Ltd.) was used for the TEM observations. Samples were dispersed in 2-propanol by an ultrasonic bath with a 10-min sonication, and a drop of the dispersion was placed on a copper grid coated with a carbon film, then dried overnight under vacuum. XRD and XPS spectra were measured using a Smart-Lab (Rigaku Corporation) and AXIS-ULTRA^DLD^ (Shimadzu Corporation), respectively, in which the binding energies were calibrated using the C 1s peak that appeared at 284.5 eV. The dual monitoring by the SEM and STEM was carried out using HF-3300 (Hitachi High Technologies) and ARM-200F (JEOL) microscopes operated at the acceleration voltage of 300 kV.

### Synthesis of s-Graphene/PyPBI

The mixture of graphite (30 mg) and PyPBI (30 mg) in DMAc (30 mL) was sonicated for 20 h, then mild centrifugation (500 g) was carried out to remove formed sedimented aggregates. The supernatant was then filtered using a PTFE membrane (0.2 μm pore size, Millipore) and washed with DMAc to remove any excess PyPBI. The obtained solid (s-Graphene/PyPBI) was dried overnight at 60 °C under vacuum.

### Loading of Au-NPs on the s-Graphene/PyPBI

To the s-Graphene/PyPBI (5.0 mg) dispersed in 60 vol % aqueous EG (10 mL), HAuCl_4_ (0.9 mg) in a 60 vol % aqueous EG solution (15 mL) and 0.1 mM of NaBH_4_ (3.0 mL) in water were added. The mixture was stirred for 24 h at room temperature under N_2_. The mixture was then filtered through a PTFE filter membrane (0.1 μm pore size, Millipore), then dried overnight under vacuum to obtain three different s-Graphene/PyPBI/Au samples.

### Electrochemical measurements

The electrochemical measurements were performed in a 0.1 M KOH solution at room temperature using an electrochemical analyzer (BAS, Model 2323). An Ag/AgCl and a platinum wire were used as the reference and counter electrodes, respectively. The ORR activity measurements were performed in an O_2_-saturated 0.1 M KOH solution using a rotating disk electrode (RDE) with a glassy carbon disk of 6-mm diameter. The metal loading on the GC electrodes was controlled at 5.3 μg_Au_ cm^−2^ for all the electrochemical experiments. The data were analyzed using the Koutecky-Levich equation. This equation was used to determine the number of electrons involved in the oxygen reductions of the s-Graphene/PyPBI/Au catalysts. The kinetic parameters were calculated using the Koutecky-Levich equation which is expressed by:


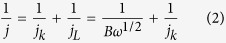



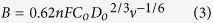



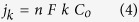


where *j* is the measured current density, *j*_*k*_ and *j*_*L*_ are the kinetic and diffusion limiting current densities, respectively, *ω* is the electrode rotation in rad/s, *n* is the overall number of electrons transferred in the oxygen reduction reactions, *F* is the Faraday constant (*F* = 96485.4 C∙mol^−1^), *C*_*O*_ is the concentration of molecular oxygen in a 0.1 M KOH solution (*C*_*O*_ = 1.2 × 10^−3^ mol∙L^−1^)^49^, *D*_0_ is the diffusion coefficient of O_2_ (*D*_*O*_ = 1.9 × 10^−5 ^cm^2^∙s^−1^)^49^, *k* is the electron transfer rate constant, and *ν* is the kinematic viscosity of the electrolyte in 0.1 M KOH (*ν* = 0.01 cm^2^ s^−1^)^50^.

## Additional Information

**How to cite this article**: Fujigaya, T. *et al.* Growth and Deposition of Au Nanoclusters on Polymer-wrapped Graphene and Their Oxygen Reduction Activity. *Sci. Rep.* **6**, 21314; doi: 10.1038/srep21314 (2016).

## Supplementary Material

Supplementary Information
