# Supplementary material for: Growth and Deposition of Au Nanoclusters on Polymer-wrapped Graphene and Their Oxygen Reduction Activity
Source: Sci Rep. 2016 Feb 22;6:21314. doi: 10.1038/srep21314 (PMC4761968; doi:10.1038/srep21314)
Supplement: Supplementary Information [file srep21314-s1.docx]

Supplementary Information

Growth and Deposition of Au Nanoclusters on Polymer-wrapped Graphene and Their Oxygen Reduction Activity

Tsuyohiko Fujigaya*^1,2^, ChaeRin Kim^1^, Yuki Hamasaki^2^, , and Naotoshi Nakashima*^1,2,3^

^1^International Institute for Carbon-Neutral Energy Research (WPI-I2CNER), Kyushu University, 744 Motooka Nishi-ku, Fukuoka 819-0395, Japan

^2^ Department of Applied Chemistry, Graduate School of Engineering, Kyushu University, 744 Motooka Nishi-ku, Fukuoka 819-0395, Japan

E-mail: fujigaya-tcm@mail.cstm.kyushu-u.ac.jp, nakashima-tcm@mail.cstm.kyushu-u.ac.jp

^3^ JST-CREST, 5 Sanbancho, Chiyoda-ku, Tokyo, 102-0075, Japan

**Table S1 | XRD analysis.** Crystal sizes of s-Graphene/PyPBI/Au calculated by Scherrer’s equation using the XRD patterns.

**Table S2 | XPS analysis.** Au composition ratio of s-Graphene/PyPBI/Au determined from XPS narrow scans shown in Figure 7b.

**Table S3 | Electrochemical analysis.** Calculated number of electrons (n) transferred during ORR for the Au-NP.

**Figure S1 | Low magnification TEM images.** Low magnification of TEM images for (a) s-Graphene/PyPBI/Au_4.5_, (b) s-Graphene/PyPBI/Au_3.3_ and (c) s-Graphene/PyPBI/Au_1.6_.

**Figure S2 | Comparison of the electron microscope images.** STEM (a) bright-field and (b) dark-field images and (c) SEM image of s-Graphene/PyPBI/Au_3.3_ at (upper) lower and (lower) high magnifications.

**Figure S3 | Effect of PyPBI layer.** (a, b) STEM images of Au-NPs directly grown on s-Graphene using 0.14 mM of HAuCl_4_.

**Figure S3 | Electrochemical analysis.** LSV curves of (a) s-Graphene/PyPBI/Au_4.5_ and (b) s-Graphene/PyPBI/Au_3.3_ on the ORR at the specified rotation rates.
